# Breastfeeding and cortisol in hair in children

**DOI:** 10.1186/s13006-021-00419-8

**Published:** 2021-10-07

**Authors:** Johnny Ludvigsson, Åshild Faresjö, Tomas Faresjö

**Affiliations:** 1 Crown Princess Victoria Children’s Hospital and Division of Pediatrics, Department of Biomedical and Clinical Sciences, Linköping University, SE-581 85 Linköping, Sweden; 2 Division of Community Medicine, Department of Health, Medicine and Care, Linköping University, SE-581 83 Linköping, Sweden

**Keywords:** Stress, Child public health, Cortisol, Hair, Development, Epidemiology

## Abstract

**Background:**

One of the most important protective health factors for children is breast-feeding, but the mechanisms for this effect are not fully elucidated. Our objective was to assess if the duration of breastfeeding influences cortisol in hair, used as a biomarker for stress in children still at school-age.

**Methods:**

ABIS (All Babies in Southeast Sweden) is a prospective population-based child cohort study of 17,055 children born Oct 1st1997- Oct 1st 1999, with the aim to study development of immune-mediated diseases. Questionnaires were answered at birth and then at regular follow-ups, and biological samples were collected. As a biomarker of stress, we measured the child’s cortisol in hair collected at 8 years of age, those randomly selected *N* = 126 children among those with enough hair samples for analyses of hair at 8 years of age. Duration of breastfeeding had been registered as well as psycho-social factors related to breastfeeding and/or stress.

**Results:**

There was a negative correlation (r = − 0.23, *p* = 0.01) between total duration of breastfeeding and hair cortisol levels at 8 years of age. In a multivariate analysis this association persisted (*p* = 0.01) even when adjusted for other potential intervening factors like age of mother at delivery and early psychosocial vulnerability in the family, an index based on 11 factors (Multivariate model: df = 5, adj R^2^ = 0.15, F = 5.38, *p* < 0.01).

**Conclusion:**

Our results show that longer breastfeeding is associated with lower cortisol levels in the child many years later. These associations should be more elaborated in further studies, and these findings also give some implications for public health. Mothers should be encouraged to breastfeed their children also in the modern society, since breastfeeding promotes health in the child. This information could be given via the obstetric departments and later at the well-baby clinics.

## Background

Stress is part of the homeostasis associated with health [1]. Transient increased stress either caused by trauma, psychosocial factors, infections etc. can usually be managed by the body [2], while repeated serious stress for instance repeated serious life events such as loss of parents (divorce, death), serious disease in the family [3] or chronic stress contribute to increased morbidity. There is no simple way to determine stress over longer time but in recent years cortisol in hair, reflecting the cortisol concentrations of the body for some months since hair grows ca 1 cm/month [4], has been used as biomarker for long-term stress both in animals [5, 6] and in humans [4, 7, 8]. Recent years, also clinical applications of cortisol measurements have emerged [9] such as biomarker of children’s stress at school and also parity-related variations in cortisol concentrations in hair during pregnancy [10, 11].

A number of factors in early childhood play an important role for health later in life. Early psychosocial stress may influence health later in adulthood [12, 13]. One of the most important health factors is breast-feeding [14, 15]. The mechanisms are regarded to be related to prevention of infections known for very long time [16], influence on the gut flora [17]), effects on the immune system related to maturation of the immune system and development of tolerance against antigens [18]. Breastfeeding with its close contact between mother and child has also profound psychological effects on the child [19]. Duration of breastfeeding is related to several factors such as age and social circumstances of the mother, smoking behavior. It is therefore difficult to know what role breastfeeding plays per se. We therefore decided to study whether breast-feeding may influence future cortisol concentration in hair used as a biomarker for stress during childhood, being an important piece of puzzle in our broader studies on the cause of autoimmune diseases, especially Type 1 diabetes, especially as both psychological stress and cortisol causing insulin resistance may play a role for development of Type 1 diabetes [3] .

## Methods

### The ABIS-study

All parents with children born between October 1st in 1997 and October 1^st^ in 1999 in Southeast Sweden (*N* = 21.700) were asked to participate in the ABIS study (All Babies in Southeast Sweden), a prospective cohort study with the aim to study how genetic and environmental factors contribute to the development of immune-mediated diseases, especially Type 1 diabetes [20]. Out of the 21.700 invited families 17.055 (78.6%) gave their informed consent to participate. Comprehensive questionnaires including up to 1000 questions were answered at birth and then at the age of 1, 3, 5, 8, 12–13 years of age, and biological samples (blood, urine, stool, hair) were collected at the follow-ups. During the first year of life a diary was used for daily registration of certain nutritional data such as time-point for introduction of different food items (among others cow’s milk protein, gluten, vegetables), infections, vaccinations, drugs and many more items (Information on questionnaires, in Swedish, can be given on reasonable request). In the questionnaires, parents were asked for duration of breastfeeding. Exclusive breastfeeding means no other food than breast milk and was based on several questions such as: “How long time from birth was the child breastfed and got only breastmilk?” which was validated by other questions about when other food was introduced. Partial breastfeeding is defined as breastfeeding in addition to formula or other food. In this analysis we focus on total breastfeeding, defined as the duration of any breastfeeding (exclusive and partial breastfeeding). The answers by the mothers have been validated by comparison with breastfeeding data registered at the well-baby clinics showing very high agreement.

In earlier follow-up of questionnaire data (from the whole ABIS cohort) one year after birth showed that 78.4% of infants were exclusively breast-fed at 3 months, 10.1% at 6 months and 3.9% up to 9 months. Partially breast-fed children were 68.9% at 6 months, while 43.6% were partially breast-fed to at least 9 months of age [21]. The median exclusive breastfeeding duration was four months and the median duration for total breastfeeding eight months. Maternal smoking, high maternal BMI, and being a single mother were associated with short-term exclusive breastfeeding. Both maternal and paternal age was positively associated with the duration of both exclusive and partial breastfeeding [22] .

In this study we analyzed a subpopulation of ABIS-children and parents since we were interested in studying different psychosocial factors impact on long-term HPA-axis activity i.e. prolonged stress measured by cortisol in hair [8]. This is an important part of the objective of the ABIS study aiming to investigate factors explaining the development of immune-mediated diseases, especially Type 1 diabetes. The 8 years follow-up cohort for ABIS-study consisted of *N* = 4031 participants. From these, we randomly selected *n* = 126 children that had sufficient hair samples to be analysed and also reliable information about breastfeeding, as well as other relevant data of the child, mother and family. All these children were included, and this number is therefore a randomly selected group from the general population. A power calculation was made estimating that the difference in hair cortisol levels for those with short breastfeeding vs. longer breastfeeding with a desired precision of 0.05 and an estimate of the true proportion of 0.40 and within a confidence interval of 0.95. This calculation gave an estimated sample of at least 96 participants, so with the selected *n* = 126 participants the study power was secured. Duration of breastfeeding was measured through a questionnaire to the mothers one year after the childbirth, when she answered about the duration in months of exclusive and partial breastfeeding. The breastfeeding variable was divided into short until 1 month, 2–6 months (medium), 7 months or longer (longer), but in the regression analyzed as a continues variable. Possible confounding factors include child’s gender, child’s birth weight (low, normal and high), gestational age (early, normal or over time), type of delivery (vaginal, Cesarean section or other problems), mother parity (first born or earlier parity), age of mother at delivery (range 18–40 years), if mother smoked during pregnancy, and a composite index of early psychosocial vulnerability in the family including eleven different indicators: single mothers, unemployment, low family income, housing conditions, low education of father or mother (i.e only primary school), parents born abroad (i.e mother or father not born in Sweden), mother experience of serious life events, mother not feeling safe or lack of social support during pregnancy, mother worried that the child could fall ill with serious disease (4).

### Measures of cortisol in hair

Trained staff cut strands of hair from the posterior vertex area of the participants’ heads in accordance with guidelines published by the Society of Hair Testing [24]. The hair was then enclosed in sealed plastic tubes marked with identification numbers and stored in room temperature until analysis. All analyses were performed at the laboratory of Clinical Chemistry at the University of Linköping. The first 3 cm of outgrowth were analyzed for cortisol concentrations using a competitive radioimmunoassay in methanol extract [24]. At least 3 mg of hair was required for reliable measurements. The samples were dissolved in radioimmunoassay buffer and analyzed. Hair samples between 3 and 10 mg were required to maintain a total inter-assay coefficient of variation below 8% for hair extraction and measurement of cortisol by the radioimmunoassay. The hair samples included in this study were analyzed 4 years after collection. Hair samples and its cortisol content has been found to be very stable over time [23]. The method is fully described elsewhere [8, 24] .

### Statistical analysis

Statistical analyses was made by using the Statistical Package for the Social Sciences software, version 23 (IBM SPSS Statistics, IBM Corporation). The measured cortisol values were logarithmised before the statistical analysis due to possible skewness in the distribution. In the univariate tests analysis of variance (ANOVA) was used for comparisons of mean. For the association between continued variables, Pearson’s correlation coefficient was applied. Variables, statistically significant in the univariate analysis, were included in a multivariate linear regression model. A *p*-value < 0.05 was considered statistically significant.

## Results

**Fig. 1:**
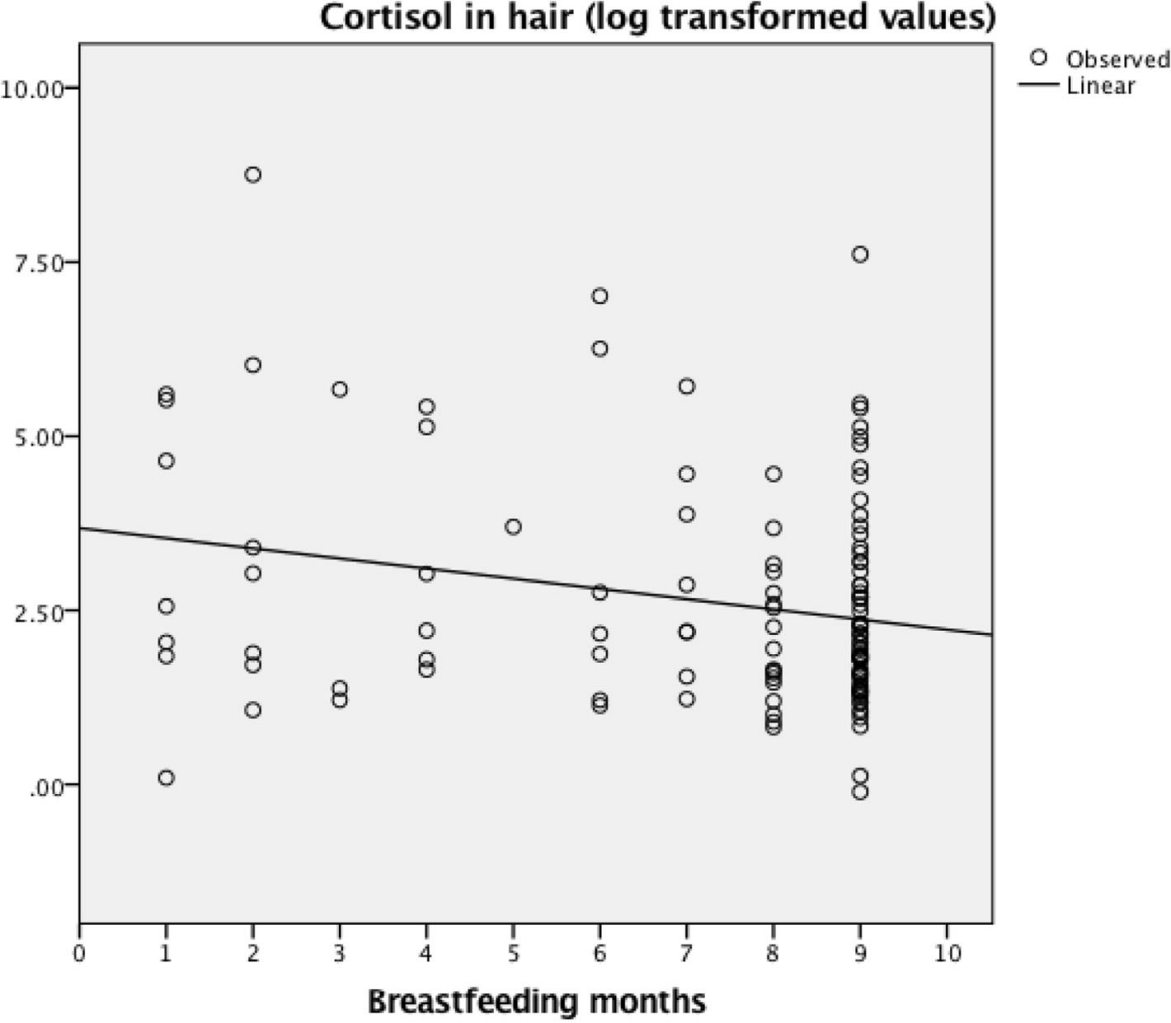
Duration of breastfeeding (months) and cortisol values (logged) among 8 year old children (N = 126)

We found an association (*p* = 0.01; r = − 0.23) between total length of breastfeeding and hair cortisol levels when the child was 8 year old. The longer the child was breastfed the lower the cortisol in hair, (Fig. 1).

The sociodemographic data and characteristics for study sample (*n* = 126) in relation to the whole cohort at 8 years follow up (*n* = 3905) is shown in Table 1. The study sample and whole cohort were quite similar in these respects only minor differences were found. Proportion of mothers, who were born abroad, was slightly higher in the study sample,and mothers in the study sample tend to be slightly older at delivery.

**Table 1:** Sociodemographic data and characteristics for the participants (*n* = 126) in relation to the whole cohort at 8 years follow up (*n* = 3905)

Variables	Participants *N* = 126		8 years cohort n = 3905		p-value
	n	%	n	%	
**Gender (children)**					0.06
Boys	56	44.4	2065	52.9	
Girls	70	55.6	1840	47.1	
**Mother education**					
Low	34	27.0	1177	30.5	0.34
Medium	44	34.9	1119	29.0	
High	48	38.1	1563	40.5	
**Father education**					
Low	40	32.8	1222	31.9	0.43
Medium	52	42.6	1469	38.3	
High	30	24.6	1142	28.9	
**Custody at 8 years**					
Mother and father	111	90.2	3443	89.9	0.90
Single parent	4	3.3	107	2.8	
Joint custody	8	6.5	279	7.3	
**Mother born abroad**					< 0.01
yes	18	14.3	191	4.9	
No	108	85.7	3678	95.1	
**Father born abroad**					0.14
Yes	10	7.9	193	5.0	
No	116	92.1	3669	95.0	
**Duration of breastfeeding**					0.22
Until 1 month	7	5.6	102	3.3	
2–6 months	22	17.7	682	21.9	
7 months or more	95	76.6	2333	74.8	
**Birth weight**					0.92
Low	12	9.5	385	10.0	
Normal	100	79.4	3089	80.0	
High	14	11.1	387	10.0	
**Gestational age**					
Early	11	8.8	310	8.1	0.97
Normal	112	89.6	3435	90.2	
Over time	2	1.6	63	1.7	
**Type of delivery**					0.83
Vaginal	104	83.2	3176	82.8	
Caesarean section	13	10.4	450	11.7	
Suction cup	8	6.4	209	5.4	
**Mother parity**					0.74
Primipara	53	42.4	1580	40.9	
Multipara	72	57.6	2281	59.1	
**Age of mother at delivery**					
16–20 years	2	1.6	29	0.7	0.02
21–35 years	98	78.4	3389	86.8	
> 35 years	25	20.0	486	12.4	
**Mother smoking during pregnancy**					0.54
Yes	6	4.8	237	6.1	
No	119	95.2	3624	93.9	
**Early psychosocial vulnerability factors in the family**					0.50
None	49	40.2	1685	45.1	
Low	49	40.2	1462	39.1	
Medium	24	19.7	578	15.5	
High	–	0.0	11	0.3	

In the univariate analysis in Table 2, it is shown that the duration of total breastfeeding was longer for older mothers (*p* = 0.01). Further, the children’s cortisol levels at eight years were higher for those with younger mothers (*p* = 0.02), also for those delivered by cesarean section or with other problems at delivery (*p* = 0.01), and if mothers were smoking during pregnancy (p = 0.01), see Table 2.

**Table 2:** Perinatal and Sociodemographic Factors and Length of Breastfeeding at Age 8 (n = 126)

	Total length of breastfeeding	
	F-value	p-value
Gender boys/girls	0.75	0.39
Birth weight (low, normal/high)	0.84	0.44
Gestational age (early, normal, over time)	1.82	0.17
Type of delivery (vaginal, section, other problems)	0.28	0.60
Mother parity (firstborn, earlier parity)	2.51	0.12
Age of mother at delivery (from 18 to 40 years	2.00	0.01
Early psychosocial vulnerability. Index of 12 variables *)	2.40	0.07
Mother smoking during pregnancy (not smoke – smoked)	0.62	0.43

*) Early psychosocial vulnerability: an index including these 12 variables: Father’s highest level of education elementary school; Mother’s highest level of education elementary school; Father unemployed or on sick leave the year before pregnancy; Mother unemployed or on sick leave during pregnancy; Living in an Apartment, as opposed to own house; Single mother; Parents born abroad; Maternal serious life event during pregnancy; Maternal lack of support during pregnancy; Mother not feeling safe during pregnancy; Mother worried over the possibility of child falling ill with serious disease; Low vs high household income

In the multivariate analysis, also described in Table 3, the association between long duration of breastfeeding and lower cortisol levels persisted (*p* = 0.01) even when adjusted for other potential intervening factors like the index of early psychosocial vulnerability in the family and age of mother at delivery. The children had also higher cortisol levels at eight years if the child was not delivered vaginally (p = 0.01) or if the mother smoked during pregnancy (*p* = 0.03).

**Table 3:** Multivariate analysis of breastfeeding and some Perinatal and Sociodemographic Factors and Hair Cortisol Concentration at Age 8

	Cortisol concentration at 8 years			
	Univariate		Multivariate*)	
	F-value	p-value	Beta	p-value
**Gender** (boys/girls)	0.32	0.57		
**Birth weight** (low, normal, high)	1.86	0.16		
**Gestational age** (early, normal, over time)	0.81	0.45		
**Type of delivery** (vaginal/section/other problems)	9.31	0.01	0.25	0.01
**Mother parity** (firstborn, earlier parity)	1.74	0.19		
**Age of mother at delivery** (from 18 to 40 years of age)	1.95	0.02	- 1.08	0.28
**Early psychosocial vulnerability in the family** (index of 12 variables)	0.25	0.86	- 0.47	0.64
**Mother smoking during pregnancy** (non-smoker, smoker)	8.62	0.01	0.19	0.03
**Duration of breastfeeding** (< 1 month - 10 months)	- 0.23 #)	0.01	- 2.82	0.01

All univariate associations measured by Anova except #) measured by Pearson correlation

*) Multivariate linear model: df = 5, adj R^2^ = 0.15, F = 5.38, p < 0.01

## Discussion

In our study we show how longer duration of breastfeeding is associated with lower cortisol in hair, a biomarker of stress, of the children at the age of 8 years, which may reflect one important mechanism behind the protective effect of breastfeeding from several diseases. We have previously shown that serious life events early in life are related to the development of diabetes-related autoantibodies [25, 26] and that serious life events during childhood increases the risk of developing Type 1 diabetes three-fold [3]. Our result therefore gives valuable information in our search for mechanisms behind the development of Type 1 diabetes. Early stress is also related to development of several other common health problems e.g. obesity [22, 27]. Breastfeeding has been found to protect against Type 1 diabetes [28] and obesity [21], with different possible explanations. One mechanism may be our finding that long breastfeeding is related to lower cortisol in children still at the age of 8, as cortisol both influences the immune system and increases insulin resistance.

### Strengths and weakness of this study

A weakness of this study is that we had only enough hair in 126 children at the age of 8 years to analyze cortisol in hair in a reliable way. The association between cortisol in hair and chronic stress exists but is of course not simple, but cortisol is known to increase insulin resistance and therefore of interest per se for the development of Type 1 diabetes. Unfortunately, among this followed group few children developed diabetes, so no further analysis was therefore possible. One could question parents own information on duration of breastfeeding, but we have data both from the diary day for day during the first year of life, and from the questionnaires answered at 1 years and 2.5 years of age, and finally we have validated these data from data in well-baby-clinics and found good agreement. This is a strength of this study as well as the design with prospective unbiased collection of data on of psychosocial factors, like serious life events without any knowledge about future development of disease or analyses of cortisol in hair.

Stress in this study is estimated using a biomarker determined with a reliable, robust and accurate method. The fact that the hair is stored for many years does not influence the method or results, and the registered data have of course not been influenced by having been kept for long time in a registry.

## Conclusion

Our results show that longer breastfeeding is associated with lower cortisol levels in the child many years later. These associations should be more elaborated in further studies, and these findings also give some implications for public health. Mothers should be encouraged to breast-feed their children also in the modern society, since breastfeeding promotes health in the child. This information could be given via at the obstetric departments and later at the well-baby clinics.

### Acknowlegements

We are grateful to all children and parents in ABIS, and thanks the funders who gave us financial support.

## Data Availability

The dataset is available from the corresponding author upon reasonable request.
